# Incipient Genetic Differentiation of the African Buffalo, *Syncerus caffer* Populations: Is Fencing Playing a Role?

**DOI:** 10.1002/ece3.71879

**Published:** 2025-07-31

**Authors:** Patrick Karanja, Johnson Kinyua, Edward Kingori, Patrick Chiyo, Vincent Obanda, Olivia Wesula Lwande

**Affiliations:** ^1^ Department of Biochemistry Jomo Kenyatta University of Agriculture and Technology (JKUAT) Nairobi Kenya; ^2^ Veterinary Service Department Kenya Wildlife Service Nairobi Kenya; ^3^ Department of Biology Duke University Durham North Carolina USA; ^4^ Institute of Primate Research Nairobi Kenya; ^5^ National Museums of Kenya Nairobi Kenya; ^6^ Department of Wildlife Health and Laboratories Wildlife Research & Training Institute Naivasha Kenya; ^7^ Department of Clinical Microbiology Umeå University Umeå Sweden; ^8^ Umeå Centre for Microbial Research (UCMR) Umeå University Umeå Sweden

**Keywords:** African ungulates, conservation, D‐loop, fencing, mtDNA, protected areas

## Abstract

Fences are increasingly used globally as a management tool in conservation to reduce wildlife depredations, disease transmission, and wildlife mortality. There are a limited number of studies on the genetic effects of perimeter fencing of protected areas on megaherbivores. Using population genetic analyses on 226 sequences of a 400 bp fragment of the mtDNA Dloop from 10 East African buffalo populations (3 fenced and 7 unfenced), the influence of spatial isolation and fencing on buffalo population genetic diversity and genetic differentiation was examined. Mean gene diversity between fenced and unfenced buffalo populations was not different (fenced: 0.978 ± 0.003, unfenced: 0.973 ± 0.004, *p* = 0.300), but nucleotide diversity was higher in fenced than unfenced populations (fenced: 0.038 ± 0.019, unfenced: 0.030 ± 0.015, *p* = 0.005). Genetic differentiation among buffalo populations based on haplotype frequencies and model‐based genetic distance was weak (FST = 0.08, ΦST = 0.06) and contributed to 6.2% and 8.5% of total genetic variance, respectively. Ninety‐three percent of population pairs were genetically differentiated by distances determined from haplotype frequencies, but only 51% of population pairs were differentiated using modeled distances, suggesting recent differentiation. There was no correlation between linearized FST and geographical distance (*r* = −0.005, *p* = 0.52), but linearized ΦST was moderately correlated with geographic distance (*r* = 0.329, *p* = 0.03). The distance effect was greater when fenced populations were excluded (ΦST: *r* = 0.464, *p* = 0.05), suggesting that insularization due to fencing is distorting isolation by distance. SSD analyses revealed that 2 of 3 fenced populations and 2 of 7 unfenced populations had non‐unimodal distributions, suggesting demographically declining populations. Our study reveals the high genetic diversity but warns that genetic erosion due to isolation, including fencing, is likely setting in and will have an impact on East African buffalo populations.

## Graphical abstract

Isolation by distance indicated that fences play a role in the genetic deterioration of confined species. The weak effects of fencing could be associated with the short period that effective fences have been in place to impact the genetics of the fenced population. The findings underscore the high genetic diversity and warn that genetic erosion due to anthropogenic activities, including fencing, is only beginning to take effect in the East African buffalo populations.

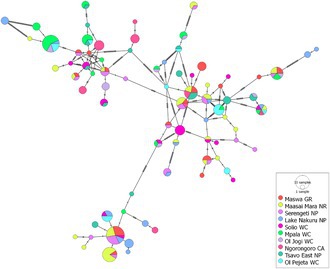

## 1 Introduction

Fences are increasingly used as a management tool in conservation to reduce wildlife depredations on crops and livestock (Cavalcanti et al. 2012; Feuerbacher et al. 2021) and along transport infrastructure bisecting wilderness areas to minimize roadkill and funnel species towards designated wildlife passages (Sawyer et al. 2012; Huijser et al. 2016). In some cases, wildlife sanctuaries are usually fenced to protect endangered species from predation and illegal harvests (Brett 1990; Smith, Conner, and Ratajczak 2020; Smith, Waddell, and Allen 2020; Koyama et al. 2023). Fencing has also been used for controlling the spread of infectious livestock diseases such as Foot‐and‐Mouth disease at the livestock‐wildlife interface (Sutmoller 2002; Fischer et al. 2011; Miller and Funston 2014; Mysterud and Rolandsen 2019).

Outside of conservation areas, fences are used to demarcate and secure international boundaries and private properties (Flesch et al. 2010; McCallum et al. 2014; Linnell et al. 2016; Pokorny et al. 2016) with inadvertent consequences for wildlife. However, fences have negative consequences as they may directly cause mortality by entangling or electrocuting species (Taylor and Rowan 1987; Boone and Hobbs 2004; Harrington and Conover 2006; Gadd 2012; Rey et al. 2012), truncate migratory routes, causing fragmentation of populations (Gadd 2012). Moreover, fencing may also reduce the carrying capacity of fenced landscapes through habitat loss and eliminating access to seasonally used foraging resources (Taylor and Rowan 1987). In some circumstances, fencing can lead to locally increased wildlife densities, magnifying the risk of dramatic population declines from density‐dependent factors such as disease spread and food limitation (Ezenwa 2004; Gadd 2012).

Genetic diversity is an essential requirement for the evolution and adaptation of populations to changing environments (Lande and Shannon 1996; Reed and Frankham 2003). This is because genetic diversity is positively correlated with resistance to diseases (Spielman et al. 2004; MacDougall‐Shackleton et al. 2005; Pearman and Garner 2005; Whiteman et al. 2006; Charpentier et al. 2008), reproductive success (McAlpine 1993; Seddon et al. 2004), growth rate (Hildner et al. 2003; Pujolar et al. 2005), increased juvenile survivorship (Da Silva et al. 2009), colonization success (Crawford and Whitney 2010) and resilience of individuals to environmental perturbations (Hughes and Stachowicz 2004; Weyrauch and Grubb Jr 2006; Oldroyd and Fewell 2007; Ehlers et al. 2008; Nevo et al. 2008). Therefore, the isolation of populations in space through fencing can eliminate gene flow, leading to increased differentiation.

Genetically distinct, small, and isolated populations are vulnerable to inbreeding and genetic drift, which can reduce genetic diversity and limit their ability to adapt to changing environments (Hepenstrick et al. 2012). Although fencing is increasingly used in wildlife management worldwide, its genetic consequences remain poorly studied, particularly in large‐bodied species such as megafauna. Most existing research has focused on the physical barrier effects of fences and their effectiveness in achieving specific management objectives. Only a few studies have investigated the genetic impacts of fencing, and these have primarily examined barriers associated with transport infrastructure (Epps et al. 2005; Kuehn et al. 2007; Hepenstrick et al. 2012; Wilson et al. 2015). For example, in California, anthropogenic barriers were found to reduce the genetic diversity of desert bighorn sheep by 15% over a span of 40 years (Epps et al. 2005).

Given that connectivity and genetic diversity are critical for the long‐term viability of wildlife populations, especially under environmental change (Shaffer 1981), there is a pressing need to better understand the genetic effects of fencing. In Kenya, some national protected areas (e.g., Nairobi National Park, Aberdare National Park, Lake Nakuru National Park) and several privately‐owned sanctuaries incorporating livestock and wildlife (e.g., Ol pejeta WC and Solio WC) are fenced.

While mitochondrial DNA (mtDNA) has been the marker of choice in many studies on broad‐scale population structure and demographic history, it is not without its limitations (Zhang and Hewitt 2003; Ballard and Whitlock 2004; Hurst and Jiggins 2005). Its effective population size is one‐quarter that of nuclear autosomal loci, resulting in faster lineage sorting and higher allele extinction rates. This can lead to an oversimplification of evolutionary relationships, underestimation of genetic diversity, increased genealogical uncertainty due to missing haplotypes, and limited resolution of more ancient population processes. In contrast, nuclear markers such as microsatellites typically offer greater resolution of recent gene flow, are inherited biparentally, and benefit from recombination, allowing for a more nuanced reconstruction of population history. However, they also present challenges, most notably the ambiguity of ancestral states due to allele homoplasy, which complicates genealogical inference (Zhang and Hewitt 2003).

Our selection of mtDNA as the primary marker in this study does not overlook these limitations, but rather reflects a strategic choice based on our study's objectives, the availability of comparative data for East African buffalo populations, and the logistical and financial constraints of developing nuclear markers at this stage. Importantly, the insights drawn from mtDNA are interpreted with a keen awareness of its limitations and in the context of its complementary role alongside nuclear data (Simonsen et al. 1998; Bertola et al. 2012). As such, this study provides a robust foundation for understanding the historical impacts of habitat fragmentation on genetic structure, while also setting the stage for future work incorporating nuclear markers to create a more comprehensive picture of gene flow and connectivity.

## 2 Methods

### 2.1 Study Populations and Sampling

**FIGURE 1:**
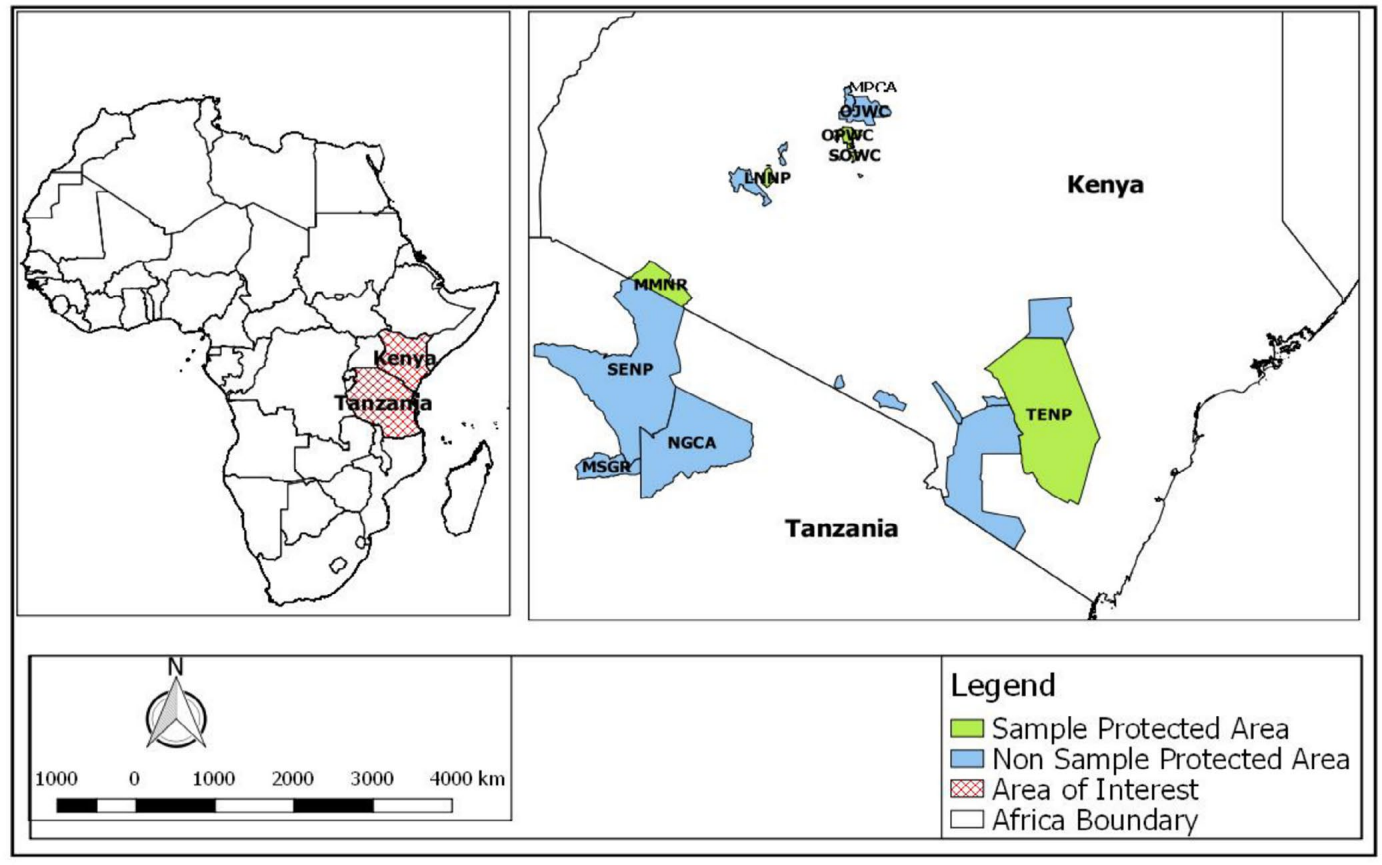
Map showing locations of the study buffalo populations in Kenya and Tanzania. Mpala Wildlife Conservancy (MPWC), Ol Jogi Wildlife Conservancy (OJWC), Maswa Game Reserve (MSGR), Ngorongoro Conservation Area (NGCA), Serengeti National Park (SENP) and Maasai Mara National Reserve (MMNR), Masaai Mara National Reserve (MMNR), Lake Nakuru National Park (LNNP), Ol Pejeta Wildlife Conservancy (OPWC), Tsavo East National Park (TENP) and Solio Wildlife Conservancy (SOWC).

We used the genetic data of buffalos from 10 different wildlife areas in Kenya and Tanzania (Figure 1), all situated within the documented range of the savannah buffalo ( *Syncerus caffer caffer* ), the only subspecies known to inhabit these regions (Heller et al. 2012; Ernest et al. 2012; Kartzinel et al. 2019). We analyzed archived blood samples from five Kenyan buffalo populations and obtained genetic data for the rest of the buffalo populations from published studies. The archived blood samples were from Maasai Mara National Reserve (MMNR), Lake Nakuru National Park (LNNP), Ol Pejeta Wildlife Conservancy (OPWC), Tsavo East National Park (TENP) and Solio Wildlife Conservancy (SOWC). The published genetic data of buffalo populations with a similar sequence length as ours or larger included Mpala Wildlife Conservancy (MPWC), Ol Jogi Wildlife Conservancy (OJWC), Maswa Game Reserve (MSGR), Ngorongoro Conservation Area (NGCA), Serengeti National Park (SENP) and Maasai Mara National Reserve (MMNR). Among these populations, LNNP, OPWC, and SOWC represent completely fenced wildlife areas, while the others are unfenced. Detailed site‐level descriptions are provided in Supporting Information S1.

In total, we had 226 sequences from the 10 populations. We generated 99 sequences from the five populations whose archived samples were sequenced in this study: LNNP (24), OPWC (17), SOWC (19), Tsavo East National Park (20), and Maasai Mara National Reserve (19). The 127 published sequences included in the study were as follows: The Mpala Wildlife Conservancy (29) and Ol Jogi Wildlife Conservancy (10) were obtained from a recent study of large mammals in Laikipia (Kartzinel et al. 2019). Further, we obtained the buffalo sequence data for Maswa Game Reserve (25), Serengeti National Park (24) and Ngorongoro Conservation area (19) from a study of buffalo genetics in the Serengeti Ecosystem (Ernest et al. 2012). Besides our Maasai Mara buffalo sequence data, we obtained additional sequences (20) for Maasai Mara National Reserve from publications (Heller et al. 2012).

The archived blood samples used in this study were collected by Kenya Wildlife Service between 2011 and 2014, during their routine disease surveillance exercises. The description of the climatic conditions and buffalo demographics in the study areas is summarized in Tables 1 and Table S2.

**TABLE 1:** Climatic and topographic variables of study buffalo populations in the protected areas in Kenya and Tanzania.

Wildlife area	Mean annual rainfall (mm)	Minimum & maximum rainfall	Rain season(s) long & short rains	Mean annual temperature	Minimum & maximum temperature	Elevation	Sources
Masaai Mara National Reserve (MMNR)	1134.8	823.8–1482.1	March–May & November–December	18°C	9.5°C–26.9°C	1480–2280	(Bartzke et al. 2018)
Ol Pejeta Wildlife Conservancy (OPWC)	702.01		March–May & November–December	24°C	10°C–28°C	1770–1820	(Wahungu et al. 2011)
Solio Wildlife Conservancy (SOWC)	750	550–900	March–May & November–December	23°C	9°C–25°C	1890–2035	(King'ori et al. 2024)
Mpala Wildlife Conservancy (MPWC)	529	296–658	April–June	24°C	12°C–36°C		(Oduor et al. 2020)
Ol Jogi Wildlife Conservancy (OJWC)	576.33	296–658	March–May & November–December	24°C	12°C–36°C	1800–1920	(Okita‐Ouma et al. 2021)
Maswa Game Reserve (MSGR)	780	600–1150	November–May	20.8°C	12.8°C–33.9°C	1600–1800	(Kimaro and Treydte 2021)
Ngorongoro Conservation Area (NGCA)	882	400–1200	March–May & October–December	15.2°C	6°C–23°C	1027–3522	(Leweri et al. 2021; Mwabumba et al. 2022)
Serengeti National Park (SENP)	855.7	550–1050	March–May & November–December	21.7°C	14°C–28°C	1000–1800	(Kilungu et al. 2017; Mahony et al. 2021)
Lake Nakuru National Park (LNNP)	869	363–1146	March–June & October–December	18°C	8.2°C–25.6°C	1760–2080	(Ng'weno et al. 2010; Ogutu et al. 2012)
Tsavo East National Park (TENP)	538	184–1201	March–May & November–January	27.9°C	22.1°C–33.6°C	150–1200	(Coe 1978; Kyale et al. 2011)

### 2.2 Genomic DNA Extraction and Amplification

Total genomic DNA was extracted from 200 μL of archived buffalo blood using the DNeasy blood and tissue kit following the manufacturers' specifications. A fragment of the left domain of the mitochondrial control (D‐Loop HVR region), adjacent to the tRNApro gene, of approximately 400 bp, was amplified by PCR, performed in a 25‐μL reaction volume containing a ready master mix (One taq 2× quickload 12.5 μL, 0.5 μL of each primer, 8.5 μL PCR water, BSA 1 μL and Template 2 μL). The primers used were 5′‐AATAGCCCCAC TATCAGCACCC‐3′ for forward and 5′‐GTGAGATGGCCCTGAAGAAA‐3′ for reverse. Amplification was performed in an Applied Biosystems Veriti 96 well Programmable thermocycler using optimization conditions of initial denaturation at 94°C for 4 min followed by 35 cycles of 40 s at 94°C, 40 s at 60°C, and 40 s at 72°C and ended by an additional 7 min extension step at 72°C. For negative controls, an equivalent volume of nuclease‐free water was substituted for DNA. PCR products were run on a 1.5% (w/v) agarose gel containing ethidium bromide at 100 V for 45 min and visualized under ultraviolet light on a trans illuminator, and positive products were visualized based on clear bands of approximately 400 bp.

### 2.3 Sequencing of PCR Products

Positive amplicons were sequenced on both sides using the primer sets targeted during amplification. Sequencing was based on the conventional Sanger method. All the sequences were manually inspected for gross anomalies and then cleaned and edited by seqtrace version 0.9.0. The sequences were aligned in MEGA X, and multiple sequence analysis (MSA) was done by clustal W and muscle software. Completed sequences were deposited to GenBank with accession numbers PQ686821 to PQ686919.

### 2.4 Genetic Diversity and Population Structure

Gene polymorphism was estimated by computing gene diversity (Hd), number of haplotypes (h), nucleotide diversity (π), number of polymorphic sites, and the average number of pairwise nucleotide differences using Arlequin 3.5 (Excoffier and Lischer 2010) and DNAsp 6 software. Statistical variation in gene and nucleotide diversity between fenced and unfenced populations and between buffalo populations was tested by performing 1000 permutations conducted using an r script adopted from Alexander et al. (2016) (Alexander 2017).

Evidence of population genetic structure was determined using two pairwise estimates of genetic differentiation, FST, derived from distance based on sharing and frequencies of haplotypes (Wright 1949) and ΦST, derived from model‐weighted genetic distances (Excoffier et al. 1992). The influence of fencing on population genetic structure was evaluated by partitioning global genetic variation between fenced and unfenced buffalo populations (FCT), within fenced and unfenced buffalo populations (FSC), and between all buffalo populations (FST). The statistical significance of these measures of genetic differentiation was performed by partitioning global genetic variability within and between populations using Analysis of Molecular Variance (AMOVA) with 10,000 permutations in the ARLEQUIN software version 3.5 (Excoffier and Lischer 2010). For ΦST, pairwise distances were calculated using the HKY85 + I + G model with the heterogeneity of the mutation rates, which are estimated to follow a gamma distribution with a shape parameter equal to 0.295661 and an invariant parameter = 0.67452 determined as the best nucleotide substitution model using PAUP software (Swofford 2003).

To test isolation by distance, the matrices of the genetic distance using linearized distance measures FST/(1 − FST) and ΦST/(1 − ΦST), (Slatkin 1995) and the natural logarithm of geographic distance (ln) between all 10 populations were compared using the Mantel test with 10,000 permutations (Mantel 1967) using the cultevo software package (Stadler 2018) in R software for statistical computing.

### 2.5 Natural Selection and Historical Demography

The evolutionary and demographic signals on the DNA sequence diversity were examined with Tajima's D and Fu's Fs neutrality test and pairwise mismatch distribution (Tajima 1989a; Slatkin and Hudson 1991; Rogers and Harpending 1992; Fu 1997), as implemented in Arlequin 3.5 (Excoffier and Lischer 2010).

Pairwise mismatch distributions were implemented to test whether a population experienced an expansion event. A goodness‐of‐fit test was used to determine the smoothness of the observed mismatch distribution (using Harpending's raggedness index, Rag) and the degree of fit between the observed and simulated data (using the sum of squares deviation, SSD) (Harpending 1994; Schneider and Excoffier 1999). The expansion signal for a population is indicated by a smooth and unimodal distribution pattern with non‐significant *p*‐values for the SSD. Rag quantifies the smoothness of the observed mismatch distribution. Low and non‐significant Rag values usually indicate an expanded population, while a significant Rag (*p* < 0.05) is evidence for rejecting the expansion model. Computation and significance estimation of both goodness of fit tests was assessed using 10,000 permutations in Arlequin 3.5 (Table 2).

**TABLE 2:** Demographic estimates and the year of the census of buffalo populations in the different conservation/protected areas in Kenya and Tanzania.

Wildlife area	Area (km^2^)	Buffalo population size	Census year	Source
Masaai Mara National Reserve (MMNR)	1530	11,604	2021	NWCR‐KWS 2021
Ol Pejeta Wildlife Conservancy (OPWC)	93	2800	2021	NWCR‐KWS 2021
Solio Wildlife Conservancy (SOWC)	70.82	701	2021	NWCR‐KWS 2021
Mpala Wildlife Conservancy (MPWC)	190	806	2021	NWCR‐KWS 2021
Ol Jogi Wildlife Conservancy (OJWC)	50	265	2021	NWCR‐KWS 2021
Maswa Game Reserve (MSGR)	2200	12,412	2014	TAWIRI 2014
Ngorongoro conservation area (NGCA)	8283	2682	2014	TAWIRI 2014
Serengeti National Park (SENP)	14,763	34,493	2014	TAWIRI 2014
Lake Nakuru National Park (LNNP)	188	6412	2021	NWCR‐KWS 2021
Tsavo East National Park (TENP)	13,747	4995	2017	Ngene et al. 2017

## 3 Results

### 3.1 Genetic Diversity and Population Structure

**FIGURE 2:**
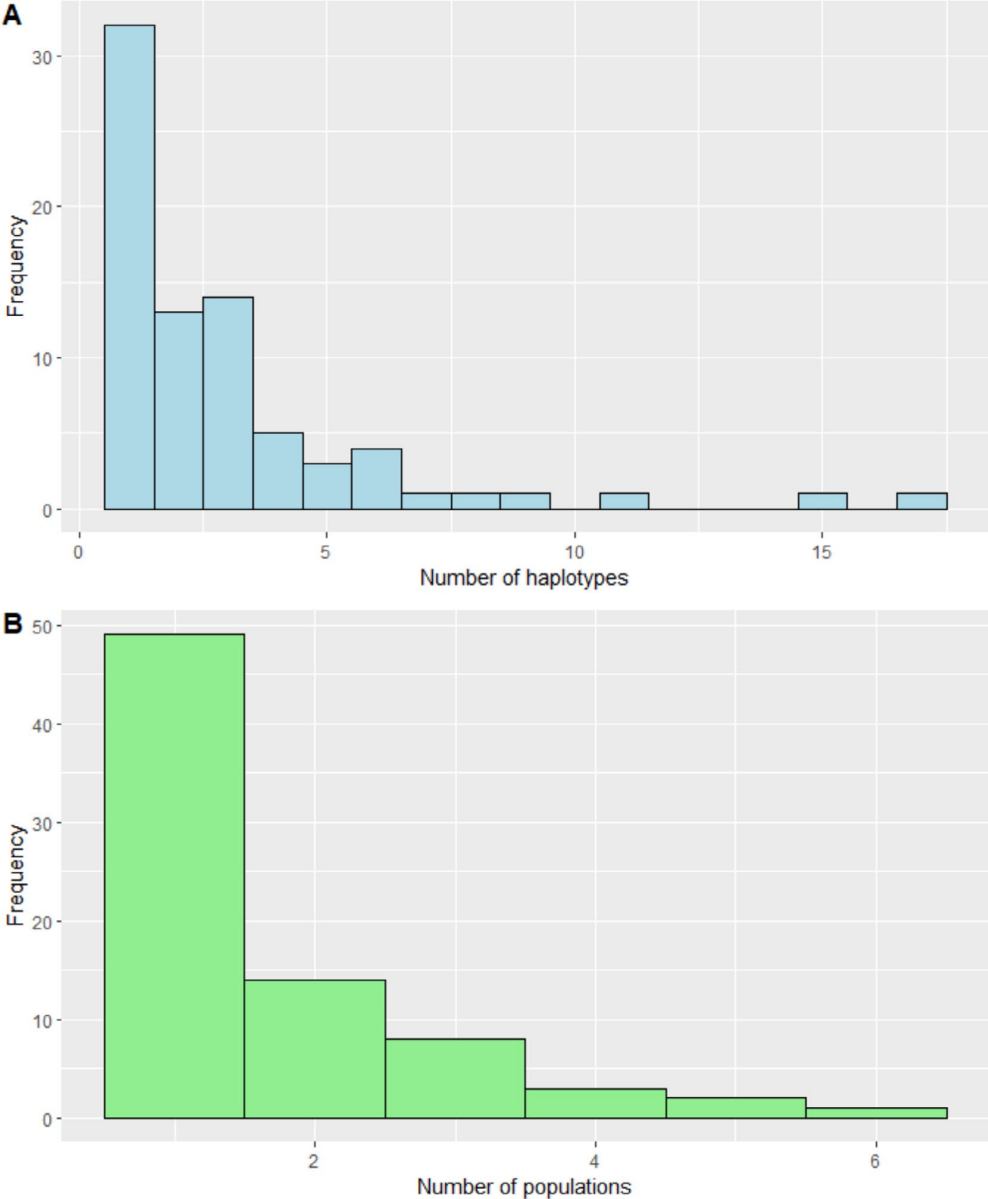
The distribution of haplotype copies (A) and haplotype sharing between the African Buffalo populations (B) in Kenya and Tanzania.

**FIGURE 3:**
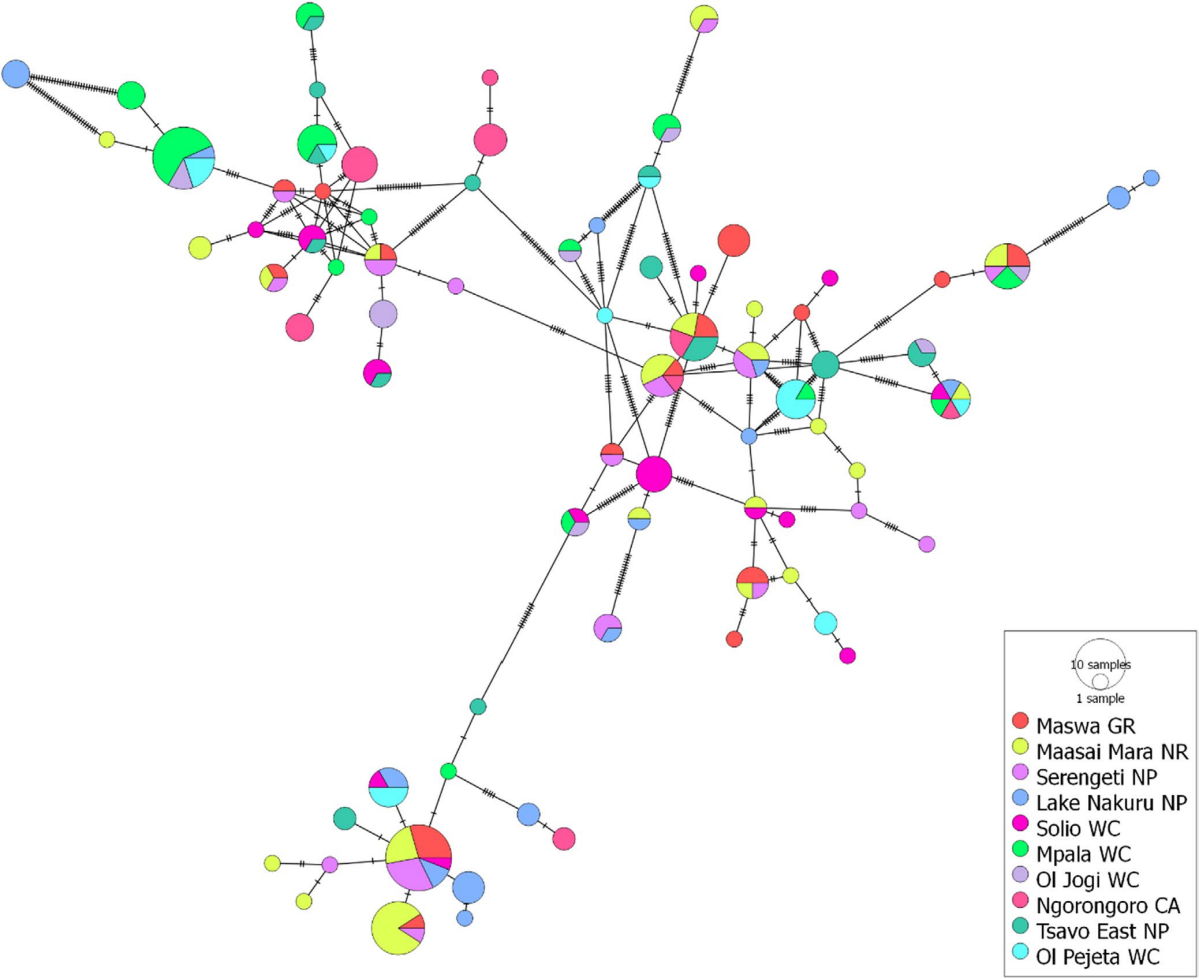
Minimum spanning haplotype network showing relationships among haplotypes in 10 buffalo populations in East Africa.

Seventy‐seven haplotypes of 496 bp in length of the mtDNA Dloop were obtained from 226 sequences originating from 10 buffalo populations in Kenya and Tanzania (Figure 1, Table 3). Most haplotypes occurred more than once (58%, *n* = 45), and the rest (42%, *n* = 32) were singletons (Figure 2, Figure S2A). Two or more populations shared 36% (*n* = 28) of haplotypes, while 64% (*n* = 49) occurred in only a single buffalo population (Figure S2B). The minimum spanning network revealed a lack of spatial sorting of haplotypes (Figure 3).

**TABLE 3:** The genetic diversity of East African buffalo populations inferred from mtDNA dloop.

Statistic	Solio WC	Ol Pejeta WC	Lake Nakuru NP	Ol Jogi WC	Mpala WC	Maasai Mara NR	Tsavo East NP	Maswa GR	Serengeti NP	Ngorongoro CA	All populations
Genetic diversity and population parameters											
Sample size, *n*	19	17	24	10	29	39	20	25	24	19	226
No. of haplotypes, h	13	8	15	7	13	21	13	15	16	8	77
Number of polymorphic (segregating) sites, S	54	46	76	45	51	68	51	41	61	44	112
Total number of mutations, Etah	54	47	78	46	52	69	52	41	63	44	116
Haplotype (gene) diversity, Hd	0.930 ± 0.047	0.875 ± 0.053	0.953 ± 0.025	0.911 ± 0.077	0.882 ± 0.045	0.933 ± 0.027	0.953 ± 0.028	0.937 ± 0.031	0.949 ± 0.031	0.877 ± 0.044	0.978 ± 0.003
Nucleotide diversity (average over loci)	0.032 ± 0.017	0.028 ± 0.015	0.045 ± 0.023	0.032 ± 0.018	0.025 ± 0.013	0.028 ± 0.014	0.027 ± 0.014	0.024 ± 0.012	0.030 ± 0.016	0.031 ± 0.016	0.032 ± 0.016
Mean number of pairwise nucleotide differences	15.427 ± 7.211	13.640 ± 6.449	21.942 ± 10.022	15.467 ± 7.557	12.345 ± 5.740	13.762 ± 6.314	13.000 ± 6.112	11.507 ± 5.397	14.609 ± 6.778	14.994 ± 7.018	15.723 ± 7.042

Gene (haplotype) diversity was generally high for both fenced and unfenced populations. Fenced buffalo populations had a mean gene diversity of 0.978 ± 0.003, and unfenced populations had a mean diversity of 0.973 ± 0.004 (Table 3). The difference in gene diversity between fenced and unfenced populations was not statistically different from chance (*p* = 0.300). However, pairwise population differences in haplotype diversity indicated that 40% (*n* = 18, *N* = 45) of unique population pairs differed significantly. Three populations with diversity at ~0.88 drove this variation: Ol Pejeta WC, Mpala WC, and Ngorongoro CA populations (Table 4). The mean number of pairwise nucleotide differences for fenced populations was 18.586 ± 8.354, and nucleotide diversity (average over loci) was 0.038 ± 0.019. For unfenced populations, the mean number of pairwise differences was 14.513 ± 6.533, while nucleotide diversity was 0.030 ± 0.015. Nucleotide diversity was higher in fenced than unfenced populations, and this difference was statistically different from that expected by chance (*p* = 0.005). Pairwise comparisons revealed that the Lake Nakuru population had the highest nucleotide diversity, significantly different from all other populations (Table S5, Table 5).

**TABLE 4:** Statistical differences in haplotype diversity between East African buffalo populations (differences are shown below diagonal, *p*‐values above diagonal).

Haplotype diversity	0.936667	0.932524	0.881773	0.877193	0.952899	0.911111	0.875	0.929825	0.949275	0.952632
	Maswa GR	Maasai Mara NR	Mpala WC	Ngorongoro CA	Lake Nakuru NP	Ol Jogi WC	Ol Pejeta WC	Solio WC	Serengeti NP	Tsavo East NP
Maswa GR	*	0.772	**0.002**	**0.004**	0.282	0.297	**0.008**	0.651	0.388	0.353
Maasai Mara NR	0.0041	*	**0.001**	**0.005**	0.142	0.389	**0.009**	0.851	0.231	0.173
Mpala WC	**0.0549**	**0.0508**	*	0.764	**< 0.001**	0.232	0.684	**0.014**	**0.001**	**0.001**
Ngorongoro CA	**0.0595**	**0.0553**	0.0046	*	**< 0.001**	0.203	0.925	**0.022**	**0.001**	**0.002**
Lake Nakuru NP	0.0162	0.0204	**0.0711**	**0.07571**	*	0.101	**0.002**	0.180	0.876	0.999
Ol Jogi WC	0.0256	0.0214	0.0293	0.03392	0.04179	*	0.205	0.422	0.120	0.129
Ol Pejeta WC	**0.0617**	**0.0575**	0.0068	0.00219	**0.07790**	0.03611	*	**0.013**	**0.002**	**0.003**
Solio WC	0.0068	0.0027	**0.0481**	**0.05263**	0.02307	0.01871	**0.05482**	*	0.244	0.220
Serengeti NP	0.0126	0.0168	**0.0675**	**0.07208**	0.00362	0.03816	**0.07428**	0.01945	*	0.842
Tsavo East NP	0.0160	0.0201	**0.0709**	**0.07544**	0.00027	0.04152	**0.07763**	0.02281	0.00336	*

*Note:* Diagonal cells marked with an asterisk (*) represent self‐comparisons and are included for structural clarity only. No statistical analysis was conducted for these entries, as intra‐population comparisons are not meaningful. Bold *p*‐values above the diagonal indicate statistically significant differences (typically *p* < 0.05), suggesting meaningful divergence in haplotype diversity. Bold values below the diagonal reflect biologically substantial differences in haplotype diversity between pairs of Buffalo population.

**TABLE 5:** Statistical differences in nucleotide diversity between populations (differences below diagonal, *p*‐values above diagonal).

Nucleotide diversity	0.023199	0.027747	0.024889	0.03023	0.044238	0.031183	0.027499	0.031103	0.029453	0.02621
	Maswa GR	Maasai Mara NR	Mpala WC	Ngorongoro CA	Lake Nakuru NP	Ol Jogi WC	Ol Pejeta WC	Solio WC	Serengeti NP	Tsavo East NP
Maswa GR	*	0.374	0.767	0.227	**0.001**	0.261	0.516	0.197	0.268	0.574
Maasai Mara NR	0.0045	*	0.534	0.656	**0.001**	0.602	0.975	0.528	0.755	0.771
Mpala WC	0.0017	0.0029	*	0.316	**< 0.001**	0.386	0.698	0.276	0.426	0.822
Ngorongoro CA	0.0070	0.0025	0.00531	*	**0.018**	0.899	0.692	0.879	0.890	0.507
Lake Nakuru NP	**0.0210**	**0.0165**	**0.0193**	**0.0140**	*	0.085	**0.008**	**0.027**	**0.011**	**0.003**
Ol Jogi WC	0.0080	0.0034	0.0063	0.0010	0.0131	*	0.608	0.992	0.794	0.469
Ol Pejeta WC	0.0043	0.0002	0.0026	0.0027	**0.0167**	0.0037	*	0.570	0.760	0.852
Solio WC	0.0079	0.0034	0.0062	0.0009	**0.0131**	0.0001	0.0036	*	0.805	0.433
Serengeti NP	0.0063	0.0017	0.0046	0.0008	**0.0148**	0.0017	0.0020	0.0017	*	0.569
Tsavo East NP	0.0030	0.0015	0.0013	0.0041	**0.0180**	0.0050	0.0013	0.0049	0.0032	*

*Note:* Asterisks (*) along the diagonal indicate self‐comparisons, included for reference only, with no statistical tests applied. Bold *p*‐values above the diagonal indicate statistically significant differences (typically *p* < 0.05), suggesting meaningful divergence in haplotype diversity. Bold values below the diagonal denotes biologically substantial differences in haplotype diversity between pairs of Buffalo population.

There was a weak and statistically significant genetic differentiation among all 10 buffalo populations (FST = 0.08, *p* < 0.001; ΦST = 0.06, *p* < 0.001), with about 6% and 8% of the molecular variance attributed to genetic variation between populations and 93% to 92% of genetic variance attributed to intra‐population genetic variance from haplotype frequency and model‐based nucleotide differences, respectively (Table 6). Fencing had limited influence on genetic differentiation, contributing to 0.24% and −0.05% of genetic variance attributed to haplotype frequency and model‐based genetic distances, respectively. This variation was not statistically different from chance expectation (Table 6).

**TABLE 6:** Molecular analyses of variance of the mtDNA dloop using haplotype frequencies and model‐based distances showing the contribution of fencing (groups) and populations (locations) to genetic variation of East African buffaloes.

Source of variation	df	Sum of squares	Variance components	Percentage of variation	Fixation indices	*p*
Genetic differentiation based on haplotype frequencies (FST)						
Among groups (FCT)	1	1.316	0.0021	0.42	0.00419	0.1994
Among populations within groups (FSC)	8	9.133	0.0304	6.17	0.06192	< 0.0001
Within populations (FST)	216	99.591	0.4611	93.42	0.06585	< 0.0001
Total	225	110.040	0.4936	100.00		
Genetic differentiation based on substitution model distance (Ф_ST_)						
Among groups (FCT)	1	0.109	−0.00002	−0.05	−0.00052	0.44673
Among populations within groups (FSC)	8	0.892	0.00340	8.51	0.08503	< 0.0001
Within populations (FST)	216	7.827	0.03623	91.55	0.08455	< 0.0001
Total	225	8.828	0.03958	100.00		

In terms of pairwise genetic differences between buffalo populations from genetic distances based on haplotype‐frequency data, there was significant genetic differentiation among all populations except three populations that shared a boundary: Maasai Mara and Serengeti, Serengeti and Maswa, and Ol Jogi and Mpala (Table 7). FST values ranged from −0.005 between Serengeti NP and Maswa GR to 0.121 between Ngorongoro CA and Ol Pejeta WC. In the case of pairwise ΦST, only 51% of population pairs were differentiated. The fixation index, ΦST ranged from 0.209 between Maswa GR and Ngorongoro CA to −0.012 between Mpala and Ol Jogi (Table S8, Table 8).

**TABLE 7:** Measures of genetic differentiation (FST) of East African buffalo populations inferred from distances calculated from haplotype frequency of the dloop of the mtDNA (FST values below diagonal, *p* values above diagonal).

	Lake Nakuru NP	Maasai Mara NR	Maswa GR	Mpala WC	Ngorongoro CA	Ol Jogi WC	Ol Pejeta WC	Serengeti NP	Solio WC	Tsavo East NP
Lake Nakuru NP	*	0.001	0.003	< 0.001	< 0.001	0.007	0.001	0.032	0.001	< 0.001
Maasai Mara NP	**0.045**	*	0.046	< 0.001	< 0.001	0.002	< 0.001	0.174	< 0.001	0.001
Maswa GR	**0.039**	**0.021**	*	< 0.001	< 0.001	0.005	< 0.001	0.609	0.001	0.001
Mpala WC	**0.070**	**0.088**	**0.086**	*	< 0.001	0.180	0.025	< 0.001	< 0.001	< 0.001
Ngorongoro CA	**0.082**	**0.084**	**0.083**	**0.119**	*	0.002	< 0.001	< 0.001	< 0.001	0.001
Ol Jogi WC	**0.058**	**0.072**	**0.068**	0.024	**0.107**	*	0.028	0.002	0.004	0.009
Ol Pejeta WC	**0.062**	**0.093**	**0.093**	**0.050**	**0.121**	**0.075**	*	< 0.001	< 0.001	< 0.001
Serengeti NP	**0.025**	0.010	−0.005	**0.082**	**0.082**	**0.064**	**0.087**	*	0.002	< 0.001
Solio WC	**0.048**	**0.061**	**0.057**	**0.092**	**0.094**	**0.074**	**0.086**	**0.050**	*	0.002
Tsavo East NP	**0.047**	**0.051**	**0.044**	**0.074**	**0.070**	**0.057**	**0.080**	**0.049**	**0.049**	*

*Note:* Diagonal entries marked with an asterisk (*) indicate self‐comparisons, included for structural reference only without statistical interpretation. Bolded entries highlight either statistically significant differentiation (*p* < 0.05), above the diagonal or biologically meaningful FST values, below the diagonal, indicating reduced maternal gene flow and possible population structuring between population pairs.

**TABLE 8:** Model based measures of genetic differentiation (ФST) of East African buffalo populations inferred from the dloop of the mtDNA (ФST values below diagonal, *p* values above diagonal).

	Lake Nakuru NP	Maasai Mara NR	Maswa GR	Mpala WC	Ngorongoro CA	Ol Jogi WC	Ol Pejeta WC	Serengeti NP	Solio WC	Tsavo East NP
Lake Nakuru NP	*	0.009	0.003	0.056	0.018	0.174	0.091	0.056	0.007	0.044
Maasai Mara NP	**0.093**	*	0.049	< 0.001	< 0.001	0.105	0.208	0.612	0.001	0.116
Maswa GR	**0.150**	**0.043**	*	< 0.001	< 0.001	0.087	0.198	0.089	0.013	0.170
Mpala WC	0.063	**0.165**	**0.195**	*	0.013	0.466	0.009	0.002	0.001	0.009
Ngorongoro CA	**0.110**	**0.167**	**0.209**	**0.083**	*	0.398	0.005	0.025	0.007	0.029
Ol Jogi WC	0.050	0.050	0.055	−0.012	0.000	*	0.344	0.426	0.161	0.803
Ol Pejeta WC	0.064	0.017	0.020	**0.125**	**0.132**	0.008	*	0.605	0.050	0.495
Serengeti NP	0.069	−0.011	0.034	**0.113**	**0.069**	−0.003	−0.012	*	0.029	0.558
Solio WC	**0.138**	**0.134**	**0.082**	**0.137**	**0.120**	0.039	**0.06**3	**0.067**	*	0.096
Tsavo East NP	**0.085**	0.028	0.021	**0.104**	**0.080**	−0.037	−0.005	−0.007	0.039	*

*Note:* Diagonal entries marked with an asterisk (*) represent self‐comparisons and are included solely for structural completeness. Values in bold indicate either statistically significant differentiation (*p* < 0.05) above diagonal or biologically notable ФST values, below diagonal, highlighting population pairs with limited maternal gene flow and possible historical isolation.

**FIGURE 4:**
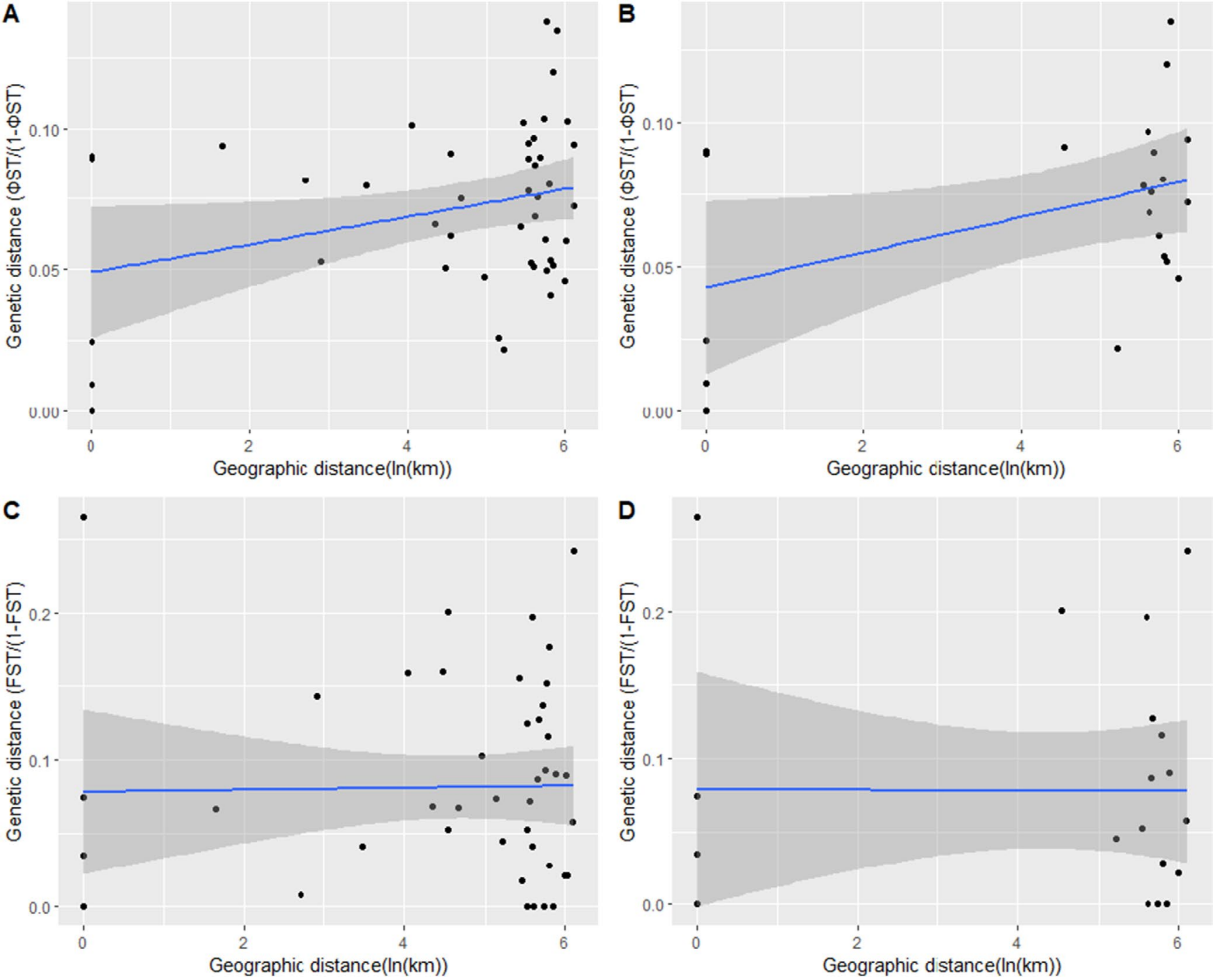
Correlations between genetic distance and spatial distance between buffalo populations in East Africa. (A) Genetic distance was determined using the HKY + G + I nucleotide substitution model on all populations (*r* = 0.329, *p* = 0.0298) and (B) excluding fenced populations (*r* = 0.464, *p* = 0.0499). In (C) genetic distance is determine from haplotype frequencies for all populations (*r* = −0.00451, *p* = 0.518) and (D) excluding fenced populations (*r* = 0.0269, *p* = 0.458).

In concordance with pairwise genetic differentiation, there was a non‐significant correlation between genetic distance and the log of linear geographical distance (Pearsons', r = −0.00451, *N* = 36, 9999 permutations, *p* = 0.518, FST, Figure 4), but model‐based genetic distance was moderately correlated with the log of geographic distance (Pearsons', *r* = 0.329, *N* = 36, 9999 permutations, *p* = 0.0298, ФST). Any genetic effects of fencing would be to reduce or weaken the isolation by distance relationships. Therefore, the effect of isolation by distance excluding fenced populations was tested. Indeed, the strength of the relationships (effect size) slightly improved when fenced populations were excluded from the analysis for both FST (Pearsons', *r* = 0.0269, *N* = 15, 720 permutations, *p* = 0.458) and model‐based genetic distance (Pearsons' *r* = 0.464, *N* = 15, 720 permutations, *p* = 0.0499).

### 3.2 The Demographic and Evolutionary History

**FIGURE 5:**
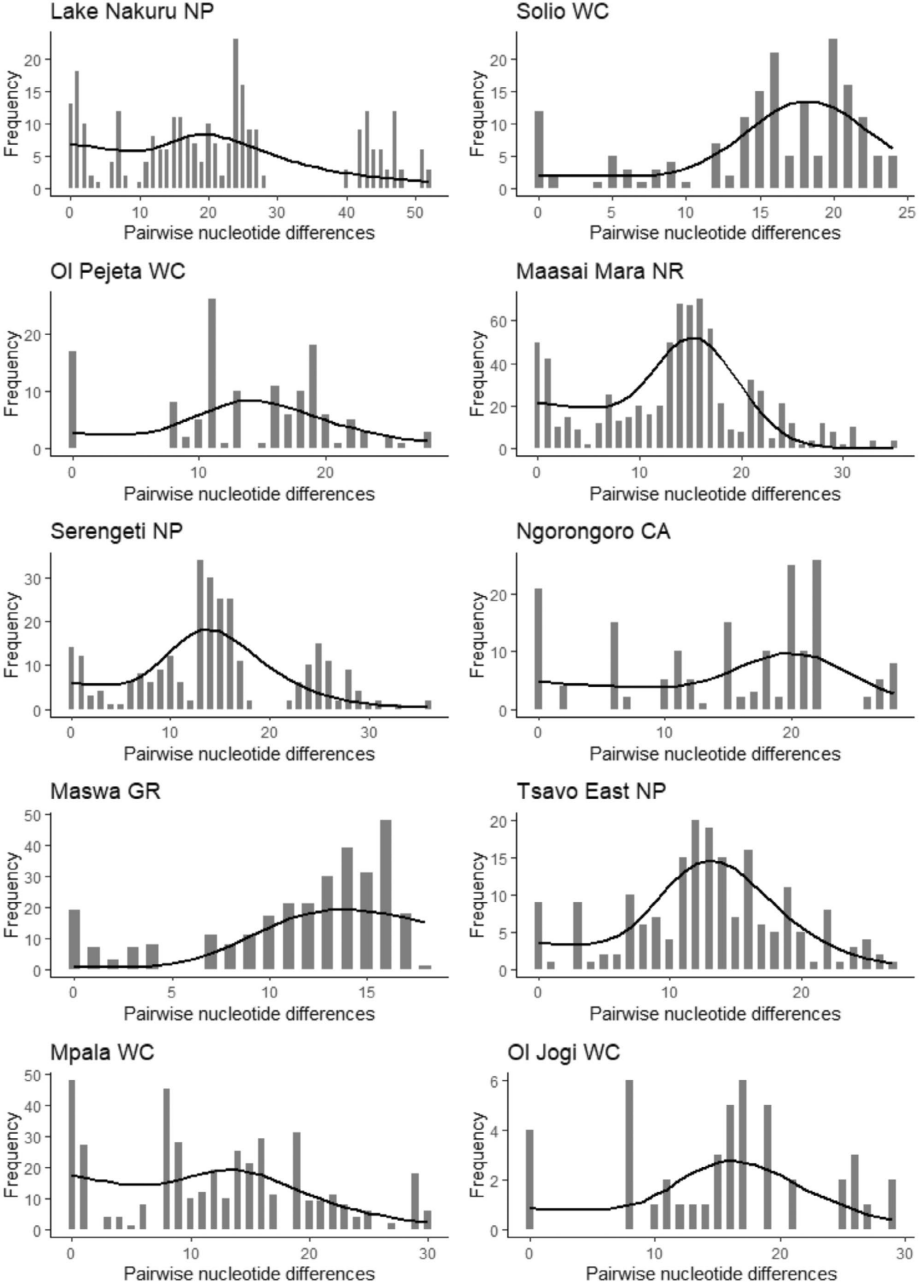
Mismatch distributions showing observed nucleotide differences (histogram) and expected differences from a population expansion model (line graph) for several buffalo populations.

The Tajima's *D* and Fu's *F*s values were not statistically different from zero, suggesting that the populations are evolving neutrally with no selection or genetic hitchhiking (Table 9). However, the distributions of the pairwise differences for two out of three fenced populations (Solio and Ol Pejeta WCs) and two of seven unfenced populations (Ngorongoro CA and Maswa WR) significantly deviated from a unimodal distribution with non‐significant SSD (Table 9, Figure 5), indicating a lack of population expansion in these populations.

**TABLE 9:** The evolutionary and demographic history of East African buffalo populations inferred from mtDNA dloop.

Statistic	Solio WC	Ol Pejeta WC	Lake Nakuru NP	Ol Jogi WC	Mpala WC	Maasai Mara NR	Tsavo East NP	Maswa GR	Serengeti NP	Ngorongoro CA	All populations
Sum of squared deviation, SSD (*p*)	**0.019 (0.043)**	**0.055 (0.003)**	0.017 (0.344)	0.047 (0.126)	0.026 (0.161)	0.011 (0.296)	0.011 (0.389)	**0.026 (< 0.0001)**	0.023 (0.055)	**0.050 (0.012)**	0.002 (0.573)
Harpending's Raggedness Index, RI (*p*)	0.041 (0.061)	**0.112 (0.005)**	0.017 (0.420)	0.105 (0.104)	0.041 (0.075)	0.012 (0.422)	0.025 (0.224)	0.024 (0.297)	0.024 (0.153)	**0.111 (0.004)**	0.002 (0.709)
Tajima's D (*p*)	−0.006 (0.583)	0.010 (0.529)	0.087 (0.597)	−0.135 (0.483)	−0.185 (0.513)	−0.523 (0.322)	−0.385 (0.366)	0.228 (0.667)	−0.413 (0.376)	0.642 (0.790)	−0.517 (0.365)
FS (*p*)	0.166 (0.539)	3.914 (0.953)	1.533 (0.773)	1.928 (0.814)	1.895 (0.814)	−0.927 (0.396)	−0.046 (0.489)	−0.604 (0.433)	−0.699 (0.418)	5.280 (0.973)	**−19.344 (0.011)**

*Note:* The values in bold indicate statistically significant results (*p* < 0.05), suggesting demographic deviations from the sudden expansion model, population structuring, or signals of historical expansion.

## 4 Discussion

### 4.1 Genetic (Gene and Nucleotide) Diversity of the East African Buffalo Populations

Our findings revealed high gene (haplotype) diversity in both fenced and unfenced buffalo populations, which was 0.978 ± 0.003 in fenced and 0.973 ± 0.004 in unfenced populations, with no significant differences between the two groups. Observed Dloop gene diversity is comparable to that of various species, including African elephant ( *Loxodonta africana* ), Asian water buffalo ( *Bubalus bubalis* ), plains' zebra ( *Equus quagga* ), and hippopotamus (*Hippopotamus amphibious*). A study of Dloop gene diversity in 9 African elephant populations revealed a mean gene diversity of 0.85 (min = 0.12 to max = 1.00) (Nyakaana et al. 2002). Similarly, Dloop diversity was reported to be 0.9174 ± 0.0157 (min = 0.5238 and max = 1.00) across 11 populations of Asian water buffalo in locations across Asia (Lau et al. 1998). In plains' zebra Dloop gene diversity assessed in 16 populations in Africa was reported to be 0.98 (min = 0 to max = 1). Continent‐wide studies have shown that African buffalo maintain high genetic diversity across their range (Van Hooft et al. 2002; Heller et al. 2012; Smitz et al. 2013). This is largely due to their ability to move over long distances, high reproductive rate, strong dispersal capacity, large population sizes, and adaptability to different types of forage (Heller et al. 2012).

Unlike gene diversity, nucleotide diversity, measured by the average number of DNA differences between pairs, was higher in fenced buffalo populations (3.8%) compared to unfenced ones (3.0%). First, the high nucleotide diversity within populations ranged from 2.4% to 4.5% and is comparable to previous findings on African buffalo genetic diversity at the D‐loop (Simonsen et al. 1998), the Asian buffalo, and similarly wide‐ranging species. For instance, nucleotide diversity for the plains zebra ( *Equus quagga* ) ranges from 0.5% to 3.3%, with an average of 2.9% (Lorenzen et al. 2008). In African savannah elephant ( *Loxodonta Africana* ), nucleotide diversity has been reported at 2.01%, ranging from 0.08% to 2.51% (Nyakaana et al. 2002). Nevertheless, the nucleotide diversity in the present study is higher than that of most wild free‐ranging herbivores. For example, the Tibetan antelope, *Pantholops hodgsonii* , has nucleotide diversity ranging from 0.4% to 1.4% (Ahmad et al. 2016), and domestic herbivores such as indigenous Tanzanian goat ( *Capra hircus* ) show high haplotype diversity (*H*_d_ = 0.9619–0.9945) but low nucleotide diversity, between 1.20% and 1.62% (Nguluma et al. 2021).

Further, the high nucleotide diversity in fenced populations compared to free‐ranging populations suggests a high effective population size, Ne, or a high mutation rate. This is because nucleotide diversity is determined mainly by mutation rate, μ and effective population size, Ne (Subramanian 2019). The presence of highly divergent haplotypes in fenced populations may indicate that ancestral population sizes were substantially larger, perhaps surpassing population sizes observed in contemporary ones. An equally plausible explanation is that the fenced populations resulted from the amalgamation of highly diverse remnant herds from different locations. The current buffalo populations in areas such as OPWC and SOWC may have originated from historically distinct and spatially separated subpopulations, such as the Aberdare ranges and Mt. Kenya regions. However, these subpopulations were likely merged into fenced populations.

### 4.2 Genetic Structure and Isolation by Distance of the East African Buffalo Populations

Genetic differentiation among buffalo populations was weak but pervasive, and only 51% (23/45) of population pairs were differentiated using ΦST, whereas 93% (42/45) of population pairs were differentiated using FST, except for contiguous populations (3/45). FST is estimated solely based on haplotype frequencies, while ΦST considers haplotype frequencies and genetic distances among haplotypes. Thus, in cases where the evolutionary time is sufficiently long for mutations to accumulate between haplotypes of isolated populations, ΦST enhances the ability to detect population structure. In contrast, when time is insufficient for haplotypes to diverge, inter‐haplotypic distances will be small, even if the frequencies of these haplotypes differ, and only FST would detect population differentiation (Sefc et al. 2007; Bird et al. 2011). Our results suggest that most of the observed genetic differentiation is recent and likely caused by the fragmentation of an originally connected population, resulting from intensive human activities that have created isolated subpopulations with little to no ongoing gene flow (Simonsen et al. 1998). This study revealed a low to moderate correlation between linearized ΦST and geographical distance (*r* = 0.329, *p* = 0.03). The distance effect was greater when fenced populations were excluded (ΦST: *r* = 0.464, *p* = 0.05). This result suggests that historical buffalo populations were large, contiguous, and in a migration‐drift equilibrium. In a large panmictic population in migration‐drift equilibrium, sub‐populations that are near each other tend to be genetically more similar than they are with more distant populations. This is because the probability of gene flow by dispersal declines with the distance between the source population and the recipient population (Slatkin 1987).

In contrast, we did not observe significant correlation between linearized FST and geographical distance among buffalo population pairs, irrespective of whether we excluded fenced populations or not. This observation suggests a more recent fragmentation of the East African buffalo population into small populations with the breakdown of the migration‐drift equilibrium. In the absence of this equilibrium, gene flow resulting from dispersal is more effective at short geographical distances, whereas random genetic drift becomes increasingly influential over larger distances (Hutchison and Templeton 1999). However, in such circumstances, the scatter in the data for population structure in paired samples increases; at high levels of variability, sampling error may be greater than genetic differentiation, and the isolation by distance (IBD) correlation disappears (Hutchison and Templeton 1999). Other than the migration‐drift equilibrium, physical anthropogenic or geographical barriers, such as rivers, mountains, fences, and human settlements, can create abrupt discontinuities in population genetic structure (Radespiel et al. 2008; Tammeleht et al. 2010; Coster and Kovach 2012) and erase or weaken the signal of genetic isolation by distance (Hartl et al. 2003; Thatte et al. 2020), a phenomenon termed isolation by resistance (McRae 2006).

### 4.3 Evolution and Demographic Trends

Tajima's D and Fu's Fs are neutrality tests that examine a population deviation from neutrality based on the expectation of constant population size at the mutation‐drift equilibrium (Tajima 1989b; Fu and Li 1993; Fu 1997). Positive Tajima's *D* values indicate fewer low and high‐frequency polymorphisms, which may suggest a recent population decline and/or balancing selection. On the other hand, negative Tajima's *D* values reflect an excess of low‐frequency polymorphisms, often indicating a selective sweep or a recent population expansion (Tajima, 1990; Joshi et al. 2013). In this study, Tajima's D and Fu's *F*s values were not statistically different from zero for all populations, suggesting they are evolving neutrally.

Past demographic history was inferred by pairwise mismatch distribution analysis between individuals by comparing the distribution of observed pairwise differences with the expected distribution in an exponentially expanding population (Schneider and Excoffier 1999). Statistical significance of the distribution was examined by comparing the sum of squared deviations (SSD) between the observed and expected pairwise mismatch distributions between sequences for each population (Harpending et al. 1993). Generally, non‐significant values of SSD indicate that the data do not deviate from that expected under the expansion model. An expansion signal is characterized by a smooth and unimodal pairwise distribution with non‐significant values for SSD (Harpending et al. 1993). (Bi)Multimodal mismatch distribution patterns point to declining population size or a structured population (Joshi et al. 2013). SSD analyses revealed that 2 of 3 fenced populations and 2 of 7 unfenced populations had bimodal or multimodal mismatch distribution patterns, pointing towards a declining population size or a structured population.

Currently, fenced protected areas tend to be in major urban areas or locations with high population density, suggesting that these fenced areas have had constrained population growth for a much longer time, driven by historical human settlements. The management practices of two unfenced parks (MSGW and NGCA) may partly explain the deviation. The practice of wildlife harvesting at MSGW, including trophy hunting, may alter age structures, skew sex ratios, and reduce effective populations (Ernest et al. 2012). The size of this park has been reduced twice since 1976 due to increased human pressure. NGCA, on the other hand, is relatively small in size, and despite the high numbers of buffalo, it permits human settlement and livestock rearing (Ernest et al. 2012).

### 4.4 Limitations

While we reaffirm the robustness of our findings, we also acknowledge two constraints. First, our analyses relied solely on mitochondrial DNA (mtDNA), which, due to its uniparental and maternal inheritance, fails to capture male‐mediated gene flow, providing a partial view of genetic diversity. Although mtDNA remains a valuable tool for assessing historical demography and broad‐scale structure in fragmented landscapes, future studies would benefit from incorporating nuclear or genome‐wide markers to validate and extend these findings (Simonsen et al. 1998; Bertola et al. 2012; Ballard and Whitlock 2004). Secondly, three of our ten study sites were fenced, reflecting the limited availability of established exclusion fences. Although this unbalanced design may reduce statistical power, it mirrors common logistical constraints in fencing research (Smith, Conner, and Ratajczak 2020; Smith, Waddell, and Allen 2020). Furthermore, early genetic erosion may occur even when diversity remains high (White et al. 2018), underscoring the need for long‐term, spatially replicated genomic studies.

## 5 Conclusion

We found limited but incipient effects of fencing on the genetics of buffaloes. Fenced populations (2 of 3) more than unfenced populations (2 of 7) had demographically declining populations. However, our findings on isolation by distance indicate that fences play a role in the genetic deterioration of confined species. The small or weak effects of fencing could be associated with the fact that effective fences have been in place for a short time to impact the genetics of the fenced population. Most fences have been in place for less than six buffalo generations (about 7.5 years each), which may be too recent for significant genetic impacts to be fully detectable. Although high mitochondrial genetic diversity was observed in fenced buffalo populations, this should not be taken as evidence that genetic erosion has not begun. Mitochondrial DNA, due to its limitations, has low sensitivity to recent demographic changes and may underestimate early losses of gene flow. The observed genetic structure and reduced haplotype sharing, however, suggest that the effects of isolation are already emerging. Therefore, current diversity likely reflects a lag before the full genetic consequences of fencing become apparent. These findings emphasize the need for continued monitoring using both mitochondrial and nuclear markers to detect and mitigate genetic erosion before it becomes irreversible.

## Author Contributions

**Patrick Karanja:** conceptualization (equal), data curation (equal), formal analysis (equal), resources (equal). **Johnson Kinyua:** conceptualization (equal), data curation (equal), formal analysis (equal). **Edward Kingori:** data curation (equal), writing – review and editing (equal). **Patrick Chiyo:** conceptualization (equal), data curation (equal), formal analysis (equal). **Vincent Obanda:** conceptualization (equal), data curation (equal), formal analysis (equal), funding acquisition (equal), project administration (equal), resources (equal), supervision (equal), writing – review and editing (equal). **Olivia Wesula Lwande:** conceptualization (equal), data curation (equal), formal analysis (equal), funding acquisition (equal), resources (equal), validation (equal), writing – review and editing (equal).

## Conflicts of Interest

The authors declare no conflicts of interest.

## Supporting information


**Data S1:** ece371879‐sup‐0001‐Supinfo.docx.

## Data Availability

The sequence data presented in this work has been deposited into GenBank (accession number PQ686821 to PQ686919).
