# Supplementary material for: Incipient Genetic Differentiation of the African Buffalo, Syncerus caffer Populations: Is Fencing Playing a Role?
Source: Ecol Evol. 2025 Jul 31;15(8):e71879. doi: 10.1002/ece3.71879 (PMC12313837; doi:10.1002/ece3.71879)
Supplement: Supplementary file 1 — Data S1: ece371879‐sup‐0001‐Supinfo.docx. [file ECE3-15-e71879-s001.docx]

**SUPPLEMENTARY MATERIALS**

**SECTION 1**

1. **STUDY SITE DESCRPTIONS**

SENP was founded in 1951 and is in the North-East of Tanzania between 34°45’–35°50’ E and 2°–3°20’ S as part of the Serengeti Ecosystem. The major vegetation types include bush grasslands, and woodlands, majorly influenced by the distribution of soil types. SENP is unfenced and completely protected, with tourism as the main human activity.

MSGR in Tanzania was established in 1962, is unfenced and lies to the southwest boundary of the SENP, between 3◦ 7’ 03.7 “S 34◦ 35’49.7” E with a mosaic of vegetation types ranging from plain grasslands, acacia woodlands, shrubs lands and thickets. The Park management permits trophy hunting, but no human settlement is allowed.

NGCA in Tanzania was established in 1958 and abuts SENP to the South-East side. It lies between latitudes -3.226162◦ and longitudes 35.4875◦. It is unfenced, and the vegetation type includes woody grasslands and thickets. It is managed as a mixed land use conservation site allowing tourism, human habitation and livestock rearing, but game harvesting is prohibited.

MPWC is a private ranch in Laikipia county in Kenya that lies north of the equator at latitudes 0° 31’ 32’’ N and longitudes 36° 53’52’’E. it was established in 1989 and is unfenced, majorly characterized by woody vegetation, including Croton dichogamous, Rhus vulgaris and Grewia species. Acacia drepanolobium is monodominant in black cotton vertisol soil, whereas Acacia brevispica, Acacia mellifera, and Acacia etbaica are dominant in the alfisol soil (Oduor et al. 2020).

OJWC is a private ranch established in 1960 in Laikipia county in Kenya and is part of the Laikipia –Samburu-Meru-Marsabit ecosystem between latitudes 0.3145033◦ and longitudes 36.9755982◦. OJWC has a rhino proof fence that allows the resident buffaloes to disperse to other areas through 18 corridors OJWC has a mosaic of lowland xeric scrub bush lands comprising of acacia and commiphora species and mesic cedar and camphor forests to the highlands.

OPWC is a private ranch in the Laikipia county, Kenya at latitude 0◦ 3’ North and longitude 36◦ 56’ East, between the foothills of Aberdare and Mount Kenya. It’s a semi-arid area with mosaic vegetation ranging from grasslands to Acacia woodlands, bushlands, and riverine woodlands. Since the 1950s, OPWC had a simple four-strand livestock fence. However, in 2006, OPWC upgraded its fence by erecting a solar-powered electric fence.

MMNR was established in 1961 and is located at latitude 1° 29’ 24” S and longitude 35° 8’ 38” E, on the South-Western part of Kenya along Kenya – Tanzania border, as part of the larger Mara ecosystem, which comprises the MMNR, the Mara triangle, and other small conservancies. Its vegetation types comprise savanna grasslands and acacia woodlands. The unfenced park allows photographic tourism only.

LNNP is located at latitudes 0° 19’ and 0° 24’S and longitude 36° 04’ and 36° 07’E. The Park was gazetted as a conservation site in 1968 and it encompassed Lake Nakuru and the adjacent habitat. In 1974, a 74-kilometre chain link fence was erected around it, except for a small section on the southern border adjoining Soysambu conservancy. Between 1986 and 1987, a high-voltage solar electric fence was constructed inside the chain link fence enclosing the entire park (Ogutu et al. 2012). The dominant vegetation includes grasslands, woodlands, and wooded-bushed grasslands.

SOWC is a private ranch in Kenya within Laikipia County, which is a very successful rhino conservation sanctuary. It’s a privately owned wildlife conservancy that was established in 1970 and is completely fenced since its establishment. It lies at latitudes 0.27°S and longitude 37°E. The major vegetation types are comprised of open glasslands, scrublands and a swamp with thousands of acacia trees.

TENP was established in 1948 and is in the South-Eastern region of Kenya and lies on latitude 2° 00’ – 3° 45’S and longitudes 38° 30’ – 39° 15’E. The park is unfenced, with the habitat mainly being open bushland with various vegetation types, including trees, grass and shrub layers. The predominant trees include Linea elata, Gravia bicolor, Acacia and Cammiphora species.

Table 2. Demographic estimates and the year of the census of buffalo populations in the different conservation /protected areas in Kenya and Tanzania

| Wildlife Area | Area (km2) | Buffalo population size | Census year | Source |
| --- | --- | --- | --- | --- |
| Masaai Mara National Reserve (MMNR) | 1,530 | 11,604 | 2021 | NWCR-KWS 2021 |
| Ol Pejeta Wildlife Conservancy (OPWC) | 93 | 2,800 | 2021 | NWCR-KWS 2021 |
| Solio Wildlife Conservancy (SOWC) | 70.82 | 701 | 2021 | NWCR-KWS 2021 |
| Mpala Wildlife Conservancy (MPWC) | 190 | 806 | 2021 | NWCR-KWS 2021 |
| Ol Jogi Wildlife Conservancy (OJWC) | 50 | 265 | 2021 | NWCR-KWS 2021 |
| Maswa Game Reserve (MSGR), | 2,200 | 12,412 | 2014 | TAWIRI, 2014 |
| Ngorongoro conservation area (NGCA) | 8,283 | 2,682 | 2014 | TAWIRI, 2014 |
| Serengeti National Park (SENP) | 14,763 | 34,493 | 2014 | TAWIRI, 2014 |
| Lake Nakuru National Park (LNNP) | 188 | 6,412 | 2021 | NWCR-KWS 2021 |
| Tsavo East National Park (TENP) | 13,747 | 4,995 | 2017 | Ngene et al, 2017 |

Table 5. Statistical differences in nucleotide diversity between populations (differences below diagonal, p-values above diagonal)

| Nucleotide diversity | 0.023199 | 0.027747 | 0.024889 | 0.03023 | 0.044238 | 0.031183 | 0.027499 | 0.031103 | 0.029453 | 0.02621 |
| --- | --- | --- | --- | --- | --- | --- | --- | --- | --- | --- |
|  | Maswa GR | Maasai Mara NR | Mpala WC | Ngorongoro CA | Lake Nakuru NP | Ol Jogi WC | Ol Pejeta WC | Solio WC | Serengeti NP | Tsavo East NP |
| Maswa GR | * | 0.374 | 0.767 | 0.227 | **0.001** | 0.261 | 0.516 | 0.197 | 0.268 | 0.574 |
| Maasai Mara NR | 0.0045 | * | 0.534 | 0.656 | **0.001** | 0.602 | 0.975 | 0.528 | 0.755 | 0.771 |
| Mpala WC | 0.0017 | 0.0029 | * | 0.316 | **<0.001** | 0.386 | 0.698 | 0.276 | 0.426 | 0.822 |
| Ngorongoro CA | 0.0070 | 0.0025 | 0.00531 | * | **0.018** | 0.899 | 0.692 | 0.879 | 0.890 | 0.507 |
| Lake Nakuru NP | **0.0210** | **0.0165** | **0.0193** | **0.0140** | * | 0.085 | **0.008** | **0.027** | **0.011** | **0.003** |
| Ol Jogi WC | 0.0080 | 0.0034 | 0.0063 | 0.0010 | 0.0131 | * | 0.608 | 0.992 | 0.794 | 0.469 |
| Ol Pejeta WC | 0.0043 | 0.0002 | 0.0026 | 0.0027 | **0.0167** | 0.0037 | * | 0.570 | 0.760 | 0.852 |
| Solio WC | 0.0079 | 0.0034 | 0.0062 | 0.0009 | **0.0131** | 0.0001 | 0.0036 | * | 0.805 | 0.433 |
| Serengeti NP | 0.0063 | 0.0017 | 0.0046 | 0.0008 | **0.0148** | 0.0017 | 0.0020 | 0.0017 | * | 0.569 |
| Tsavo East NP | 0.0030 | 0.0015 | 0.0013 | 0.0041 | **0.0180** | 0.0050 | 0.0013 | 0.0049 | 0.0032 | * |

Table 8. Model based measures of genetic differentiation (ФST) of East African buffalo populations inferred from the dloop of the mtDNA (ФST values below diagonal, p values above diagonal)

|  | Lake Nakuru NP | Maasai Mara NR | Maswa GR | Mpala WC | Ngorongoro CA | Ol Jogi WC | Ol Pejeta WC | Serengeti NP | Solio WC | Tsavo East NP |
| --- | --- | --- | --- | --- | --- | --- | --- | --- | --- | --- |
| Lake Nakuru NP | * | 0.009 | 0.003 | 0.056 | 0.018 | 0.174 | 0.091 | 0.056 | 0.007 | 0.044 |
| Maasai Mara NP | **0.093** | * | 0.049 | <0.001 | <0.001 | 0.105 | 0.208 | 0.612 | 0.001 | 0.116 |
| Maswa GR | **0.150** | **0.043** | * | <0.001 | <0.001 | 0.087 | 0.198 | 0.089 | 0.013 | 0.170 |
| Mpala WC | 0.063 | **0.165** | **0.195** | * | 0.013 | 0.466 | 0.009 | 0.002 | 0.001 | 0.009 |
| Ngorongoro CA | **0.110** | **0.167** | **0.209** | **0.083** | * | 0.398 | 0.005 | 0.025 | 0.007 | 0.029 |
| Ol Jogi WC | 0.050 | 0.050 | 0.055 | -0.012 | 0.000 | * | 0.344 | 0.426 | 0.161 | 0.803 |
| Ol Pejeta WC | 0.064 | 0.017 | 0.020 | **0.125** | **0.132** | 0.008 | * | 0.605 | 0.050 | 0.495 |
| Serengeti NP | 0.069 | -0.011 | 0.034 | **0.113** | **0.069** | -0.003 | -0.012 | * | 0.029 | 0.558 |
| Solio WC | **0.138** | **0.134** | **0.082** | **0.137** | **0.120** | 0.039 | **0.06**3 | **0.067** | * | 0.096 |
| Tsavo East NP | **0.085** | 0.028 | 0.021 | **0.104** | **0.080** | -0.037 | -0.005 | -0.007 | 0.039 | * |


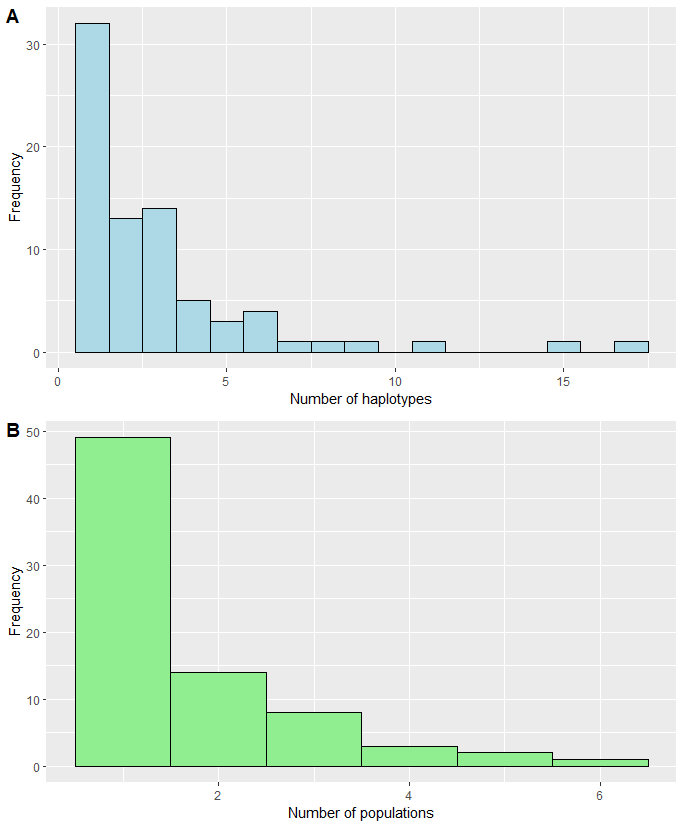


Figure 2. The distribution of haplotype copies (A) and haplotype sharing between the African Buffalo populations (B) in Kenya and Tanzania
